# New Autoantibody Specificities in Systemic Sclerosis and Very Early Systemic Sclerosis

**DOI:** 10.3390/antib10020012

**Published:** 2021-03-28

**Authors:** Roberto Lande, Raffaella Palazzo, Anna Mennella, Immacolata Pietraforte, Marius Cadar, Katia Stefanantoni, Curdin Conrad, Valeria Riccieri, Loredana Frasca

**Affiliations:** 1 Istituto Superiore di Sanita’, National Centre for Pre-Clinical and Clinical Drug Research and Evaluation, Pharmacological Research and Experimental Therapy Unit, 00166 Rome, Italy; roberto.lande@iss.it (R.L.); raffaella.palazzo@iss.it (R.P.); anna.mennella@guest.iss.it (A.M.); 2 Department of Dermatology, University Hospital CHUV, 1011 Lausanne, Switzerland; curdin.conrad@chuv.ch; 3 Department of Oncology and Molecular Medicine, Istituto Superiore di Sanità, 00161 Rome, Italy; immacolata.pietraforte@iss.it; 4 Dipartimento di Scienze Cliniche e Internistiche, Anestesiologiche e Cardiovalscolari, University Sapienza, 00185 Rome, Italy; marius.cadar@uniroma1.it (M.C.); katia.stefanantoni81@gmail.com (K.S.); valeria.riccieri@uniroma1.it (V.R.)

**Keywords:** autoimmune diseases, chemokine (C-X-C motif) ligand 4 (CXCL4), CXCL4-L1, autoantibodies, Systemic Sclerosis, Very Early Diagnosis of Systemic Sclerosis (VEDOSS), IFN-I signature, biomarkers

## Abstract

Chemokine (C-X-C motif) ligand 4 (CXCL4) is a biomarker of unfavorable prognosis in Systemic Sclerosis (SSc), a potentially severe autoimmune condition, characterized by vasculitis, fibrosis and interferon (IFN)-I-signature. We recently reported that autoantibodies to CXCL4 circulate in SSc patients and correlate with IFN-α. Here, we used shorter versions of CXCL4 and CXCL4-L1, the CXCL4 non-allelic variant, to search for autoantibodies exclusively reacting to one or the other CXCL4 form. Moreover, to address whether anti-CXCL4/CXCL4-L1 antibodies were present before SSc onset and predicted SSc-progression, we longitudinally studied two VEDOSS (Very Early Diagnosis of Systemic Sclerosis) patient cohorts, separating SSc-progressors from SSc-non-progressors. We found that anti-CXCL4-specific autoantibodies were present in both SSc and VEDOSS patients (both SSc-progressors and SSc-non-progressors). Anti-CXCL4-L1-specific autoantibodies were especially detected in long-standing SSc (lsSSc). Anti-CXCL4/CXCL4-L1 antibodies correlated with IFN-α and with specific SSc-skin features but only in lsSSc and not in early SSc (eaSSc) or VEDOSS. Thus, a broader antibody response, with reactivity spreading to CXCL4-L1, is characteristic of lsSSc. The early anti-CXCL4 autoantibody response seems qualitatively different from, and likely less pathogenic than, that observed in advanced SSc. Lastly, we confirm that anti-CXCL4 autoantibodies are SSc-biomarkers and uncover that also CXCL4-L1 becomes an autoantigen in lsSSc.

## 1. Introduction

Systemic Sclerosis (SSc) is an autoimmune disease characterized by three hallmarks: autoimmunity, fibrosis and vasculopathy [1]. Autoreactive T-cells and autoantibodies expand and participate to SSc pathogenesis; thus, the autoimmune component is of importance in the disease [1,2,3,4]. We can distinguish two major SSc forms: limited cutaneous (lcSSc) and diffuse cutaneous (dcSSc) SSc, and depending on the disease duration, we identify an early (eaSSc, disease duration <5 years) or long-standing SSc (lsSSc, disease duration >5 years) [1,5]. Dysregulation of the innate immune system in genetically predisposed individuals and aberrant Toll-like receptor (TLR) activation, are likely involved in SSc pathogenesis [3,6].

C-X-C motif ligand 4 (CXCL4) is an SSc biomarker up-regulated in the skin and circulation of SSc patients and is associated with worse disease prognosis [7,8]. CXCL4 is linked to the type I interferon (IFN-I) signature, which is usually present in 50% of SSc patients [1]. In turn, an IFN-I signature is also linked to a poor SSc prognosis [9,10,11].

Recently, we dissected the mechanistic link between CXCL4 and IFN-α, and plasmacytoid dendritic cells (pDCs) activation, the major IFN-I producing cells in the body [12]. We demonstrated that CXCL4 enables innate immune-recognition of natural DNA by pDCs, by forming liquid nanocrystals, which protect the bound DNA from enzymatic degradation and facilitate up-take by pDCs. In such periodic nanocrystals, DNA ligands are organized in molecular structures that induce optimal TLR9-driven IFN-I secretion by pDCs [12]. The CXCL4 capacity to condense DNA/RNA in nanocrystalline structures could facilitate anti-CXCL4 autoantibody generation in SSc, as particulate structures confer antigenicity to otherwise poorly immunogenic molecules [13,14], and in fact, we have demonstrated that CXCL4 acts as an autoantigen in a consistent proportion of SSc patients. Most importantly, anti-CXCL4 autoantibodies correlate with the IFN-α signature [15].

CXCL4 has a non-allelic variant, called CXCL4-L1, which unlike CXCL4 is produced by smooth cells of blood vessels [16]. CXCL4-L1 concurs to pathogenesis of diseases where platelets activation plays a pivotal role [16,17,18,19,20]. SSc platelet activation has long been considered important in the pathogenesis [20]. Notably, CXCL4 and CXCL4-L1 are both contained into the platelets’ a-granules and are released together during platelets activation. For example, both CXCL4 and CXCL4-L1 can be found upregulated in a condition called phospholipid syndrome (APS), in which platelets activation occurs [19]. Thus, it is likely that CXCL4-L1 plays a role in SSc, besides CXCL4, for instance as a result of its strong anti-angiogenic properties [16]. If CXCL4 is upregulated, it is highly likely that CXCL4-L1 reaches high concentration in the SSc blood too, and indeed, a preliminary communication has shown that CXCL4-L1 is upregulated in blood of SSc patients, as compared to normal subjects [21]. At present, whether CXCL4-L1 is also an autoantibody target in SSc remains unknown.

Patients with “Very early diagnosis of systemic sclerosis” (VEDOSS) experience the Raynaud’s phenomenon, and are positive for the specific SSc-autoantibodies, namely, anti-topoisomerase (ATA) and anti-centromere (ACA) antibodies [22,23]. Some VEDOSS patients progress to SSc but others do not, and it will be important to discover specific biomarkers that distinguish patients at risk of progression from possible non-progressors, among VEDOSS cases, to timely start appropriate therapies. Here, we have tried to understand what is the distribution of anti-CXCL4 and anti-CXCL4-L1 antibody reactivity in SSc (either eaSSc or lsSSc) and VEDOSS patients (either SSc-progressors and SSc-non-progressors), to indentify new biomakers of disease/disease progression and new players in SSc pathogenesis.

## 2. Materials and Methods

### 2.1. Human Studies and Samples

Blood samples (from 1 to 3 mL) from SSc and VEDOSS were obtained in Rome, Italy, Policlinico Umberto I. Plasma or sera from HD, matched for age and sex with SSc as much as possible, were from the blood centers at Policlinico Umberto I, Italy. SSc patients satisfied the American College of Rheumatology (ACR)/European League Against Rheumatism (EULAR) 2013 classification criteria [24]. To corroborate data, we used two different VEDOSS cohorts: For the discovery cohorts, we disposed of plasma, and for the replication cohort, we disposed of sera. Plasma was obtained from whole blood collected in Vacutainer EDTA tubes (Becton and Dickinson, Franklin Lakes, NJ, USA), to avoid clotting. One milliliter of blood was then centrifuged at 2000× *g* for 15 min. The supernatant was collected with a pipette and stored in 2 mL tubes at −80 °C for future experiments. Serum was obtained from whole blood allowed to clot at room temperature. The tube with the clot was then centrifuged at 2000× *g* for 15 min, and the supernatant was collected with a pipette and also stored at −80 °C. Small aliquots of plasma and sera were prepared to avoid freeze-thaw cycles. Exclusion criteria included patients treated with biologics.

We obtained all samples upon approval by Ethic Committees of University Sapienza (rif.1725, rif.2125, IT). All blood donors gave informed consent according to the Helsinki’s declaration.

### 2.2. Antigens

Human recombinant CXCL4 was from Sino Biological (Beijing, China). Both CXCL4 and CXCL4-L1 were also synthesized by Biomatik (Kitchener, ON, Canada), as reported [12]. The COOH-terminal part of CXCL4 and CXCL4-L1 were purchased from Phoenix France, S.A.S. These peptides represent the last 27-amino acids at the COOH-terminal of the CXCL4 and CXCL4-L1 molecules [25].

### 2.3. IFN-α Determination in Sera/Plasma

IFN-α levels in blood were detected by enzyme-linked immune sorbent assay (ELISA), using the MabTech kit (Cincinnati, OH, USA), as described [12]. Sera and plasma were diluted 1:4 in phosphate buffer solution (PBS 1×).

### 2.4. ELISA for Anti-CXCL4/CXCL4-L1-Autoantibodies Determination in Sera/Plasma

We measured the anti-CXCL4 and anti-CXCL4-L1 antibodies by ELISA, as described [15]. Briefly, 96-well flat-bottom plates (non-binding surface polystyrene, Corning, Corning, NY, USA) were coated with 2 µg/mL CXCL4, or CXCL4 L1, or with short CXCL4/CXCL4-L1 27-mer peptides (all at the same mMolar concentrations as CXCL4/CXCL4-L1) in carbonate buffer (0.1 M NaHCHO3, pH 9), for 2 h (or overnight), and subsequently washed four times with PBS 1× + 0.1% Tween-20. This washing buffer was used for washing at all steps. Blocking buffer, containing 2% bovine serum albumin (BSA, Sigma-Aldrich, St. Louis, MO, USA) in PBS 1x was used for at least 1 h (or overnight) to saturate unspecific binding sites. After washing, sera/plasma were diluted at various concentrations (usually at 1:100 or 1:200) in PBS + 2% BSA, followed by an 1 h of incubation with a horseradish peroxidase (HRP)-conjugated goat anti-human IgG (Sigma-Aldrich, St. Louis, MO, USA), (dilution 1:5000 in PBS). The color was developed for 5 min with 3,3′,5,5′-tetramethylbenzidine (TMB) substrate (Sigma-Aldrich). The reaction was stopped by adding 50 µL of 2N H_2_SO_4_, and absorbance was determined at 450 nm, with a reference wavelength of 540 nm. Anti-CXCL4/CXCL4-L1 antibodies were considered positive and significant when they exceed the mean OD values obtained with HD, plus two standard deviations (SD).

### 2.5. Statistical Analyses

We assessed differences between mean values by Mann–Whitney’s test (one tailed or two tailed). Statistical significance was set at *p* < 0.05. Correlation analyses were performed by Spearman’s rank correlation tests. Data were analyzed, and correlations were calculated, using GraphPad Prism 7.0 (GraphPad Softwer, San Diego, CA, USA).

## 3. Results

### 3.1. SSc and VEDOSS Can Share Autoantibody Specificity

**Figure 1:**
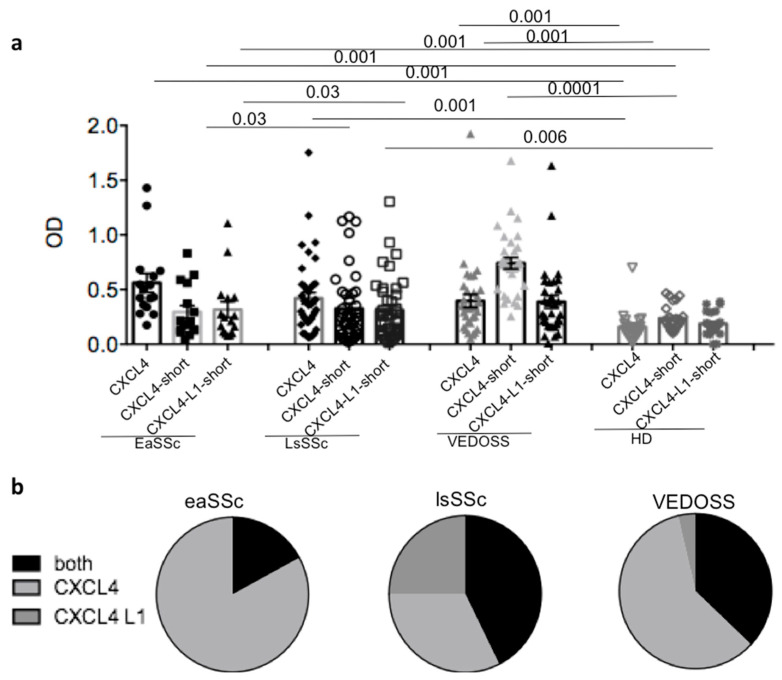
Systemic Sclerosis (SSc) and Very Early Diagnosis of Systemic Sclerosis (VEDOSS) share autoantibody specificity. (**a**) SSc (early SSc (eaSSc) and long-standing SSc (lsSSc)), control healthy donors (HD) and VEDOSS patients were tested for serum or plasma antibody reactivity to entire Chemokine (C-X-C motif) ligand 4 (CXCL4) (CXCL4) or to the 27 mer peptides spanning the COOH-term of CXCL4 (CXCL4-short) or CXCL4-L1 (CXCL4-L1-short), by ELISA. Results are reported as optical density (OD). Horizontal bars represent the mean; vertical bars are standard error of the mean (SEM); *p* values by Mann–Whitney test. Cut-off lines for anti-CXCL4 (continuous black line), for CXCL4-COOH (dotted gray line) and for anti-CXCL4-L1 (dotted black line) antibodies are reported on the graph. (**b**) Anti-CXCL4 and anti-CXCL4-L1 antibody reactivity distribution in SSc and VEDOSS, represented as cake diagrams. In the diagrams, the reactivity to one or the other CXCL4 form is reported as percent of reactivity (entire diagram represents 100% of the clinical samples tested).

**Figure 2:**
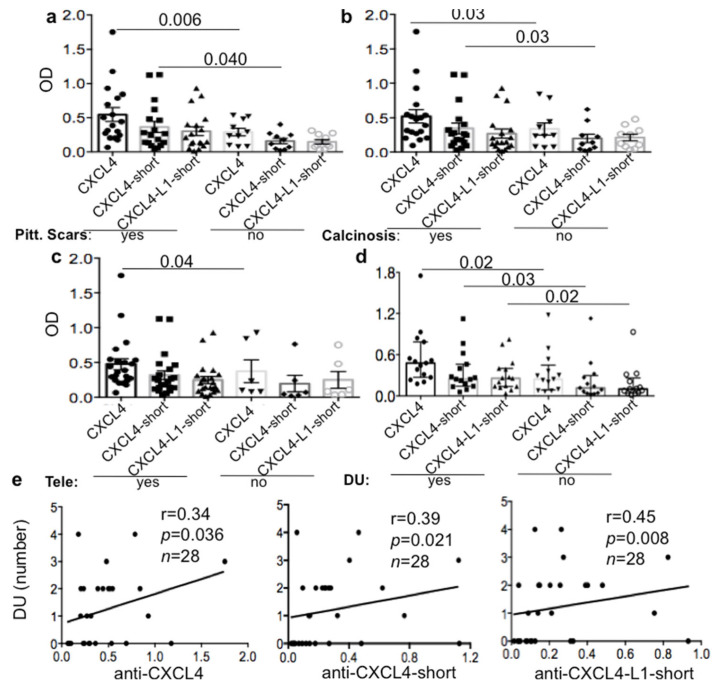
Anti-CXCL4 and/or CXCL4-L1 antibodies are higher in lsSSc patients with skin involvement. LsSSc patients with pitting scars (**a**), calcinosis (**b**), telangectasia (**c**) and digital ulcers (DU) (**d**) were tested for antibody reactivity to entire CXCL4 (CXCL4) or to the 27 mer peptides spanning the COOH-term of CXCL4 (CXCL4-short) or CXCL4-L1 (CXCL4-L1-short), by ELISA. Results are reported as optical density (OD). Horizontal bars represent the mean, vertical bars are standard errors of the mean (SEM) in a–c and median plus interquartile range in (**d**), *p* values by Mann–Whitney test. (**e**) Number of DU in lsSSc plotted against anti-CXCL4 (entire CXCL4) antibodies or antibodies directed to the COOH-part of CXCL4 (anti-CXCL4-short) or anti-CXCL4-L1 (anti-CXCL4-L1-short). Spearman “r” coefficient, *p* values and sample size *n* are indicated.

To address the presence of anti-CXCL4 and anti-CXCL4-L1 antibodies in SSc, as compared to VEDOSS, we took advantage of an in house-ELISA test that we had previously set-up [15]. As control, we assessed the anti-CXCL4/CXCL4-L1 antibody reactivity in healthy donors (HD). (See Table 1, for SSc patients, VEDOSS patients, and control HD studied). In keeping with previously published work, anti-CXCL4-autoantibody reactivity was detectable in SSc and not in HD (Figure 1a) [15]. SSc subtype analysis indicated that anti-CXCL4 autoantibodies were present in both eaSSc and lsSSc. Moreover, anti-CXCL4 antibody responses were present in VEDOSS patients. To address whether anti-CXCL4 autoantibodies reacted uniquely to the wild type (wt) CXCL4 or to the CXCL4 non-allelic variant CXCL4-L1, differing from the wt CXCL4 by three amino acid substitutions at the very COOH-term, we differentiated among patients that responded to both molecules or to either one or the other form. To do so, we used short-COOH forms of CXCL4 and CXCL4-L1, spanning the 27 amino acid sequence at the COOH-terms of CXCL4/CXCL4-L1, as antigen in ELISA tests. When the patients responded to the entire CXCL4 and/or to the short form of CXCL4, but not to the short form of CXCL4-L1, we considered them only reacting to the wt molecule. Instead, when reactivity to all three antigens was present, we considered the autoantibodies specific for both CXCL4 and CXCL4-L1. In case the patients responded to the COOH-part of CXCL4-L1 without any reactivity to the short form of wt CXCL4, we considered those patients as exclusively reacting to CXCL4-L1. Five out of 16 (31%) eaSSc, and 13 out of 41 (32%) lsSSc patients, respectively, responded to CXCL4 (Figure 1a). Seven out 33 VEDOSS (21%) responded to CXCL4. VEDOSS mainly responded to the COOH-part of CXCL4 (17 out of 33, 52%). Four out of 33 VEDOSS also responded to CXCL4-L1 (12%). In Figure 2b, the cake diagrams show the distribution of reactivity to CXCL4 and CXCL4-L1 in all groups. An antibody response exclusively directed to CXCL4-L1 was mostly present in lsSSc, whereas eaSSc and VEDOSS recognized predominantly the wt CXCL4 or reacted to both forms. These results suggest that the antibody response of lsSSc patients is broader than that present before disease onset or the response detected at early SSc stages.

### 3.2. Anti-CXCL4 Autoantibodies Can Be Associated with Skin Involvement in lsSSc

We next sorted the SSc patients on the bases of their different cutaneous manifestations. The antibody response directed to CXCL4 was significantly higher in lsSSc patients with skin involvement, such as typical skin scars called pitting scars, calcinosis (a deposition of insoluble calcium in the skin, associated with longer disease duration), telengiectasia and digital ulcers (DU) (Figure 2a–d) but only in lsSSc [26,27,28]. Both anti-CXCL4 and anti-CXCL4-L1 autoantibodies correlated with digital ulcers (DU) in lsSSc patients (Figure 2e). In contrast, anti-CXCL4 antibody reactivity and number of DU inversely correlated in eaSSc (Figure S1). LsSSc patients with overt lung fibrosis (which was associated with a higher disease duration) were also the ones with a higher antibody response to CXCL4 (Figure S2). In eaSSc, we did not find any association and/or correlation between anti-CXCL4 antibodies and the above reported disease parameters. In contrast, the eaSSc patients with pitting scars were characterized by lower anti-CXCL4 antibody reactivity, as compared to those with no pitting scars (Figure S3). These results suggest that anti-CXCL4 autoantibodies in lsSSc, but not in eaSSc, may be markers of skin inflammation, in addition to being associated with lung fibrosis, as previously reported [15]. The results seem also suggest that anti-CXCL4-, and possibly anti-CXCL4-L1-autoantibodies, may acquire pathogenic functions in the skin inflammation and lung fibrosis at late SSc-phases.

### 3.3. Anti-CXCL4/CXCL4-L1 Autoantibodies Correlate with IFN-I in lsSSc but Not in eaSSc/VEDOSS

**Figure 3:**
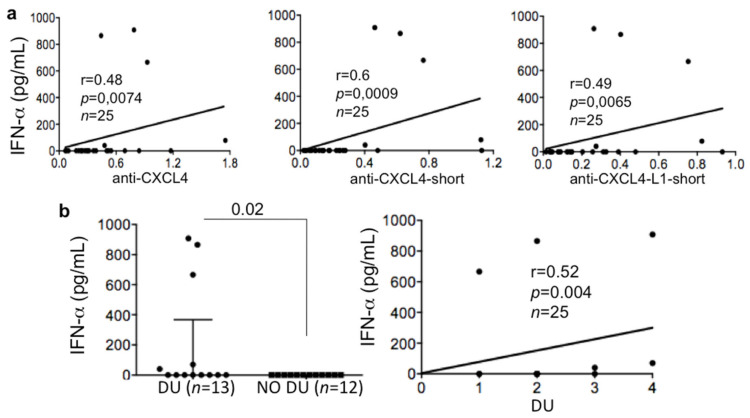
Anti-CXCL4/CXCL4-L1 antibodies correlate with IFN-a in lsSSc. (**a**) Amounts of IFN-a in sera or plasma, pg/mL, measured by ELISA, plotted against anti-CXCL4 (entire CXCL4) antibodies or antibodies directed to the COOH-part of CXCL4 (anti-CXCL4-short) or anti-CXCL4-L1 (anti-CXCL4-L1-short) expressed as OD. (**b**, left panel) LsSSc patients were divided in two groups, one presenting DU and the other without DU (NO DU). IFN-a was measured in both groups by ELISA as in (**a**). Horizontal bars are the medians plus interquartile range; *p* value by Mann–Whitney’s test. (**b**, right panel) Level of plasma IFN-a plotted against number of DU in lsSSc patients. Spearman “r” coefficient, *p* values and sample size, N, are indicated.

In our previous study, we have shown that anti-CXCL4 autoantibodies correlated with IFN-α measured in SSc blood (sera or plasma) by ELISA assay [15], in two SSc cohorts. Here, we found again a correlation between the anti-CXCL4 antibody reactivity and IFN-a in a new cohort but only in lsSSc patients (Figure 3a) (r = 0.048, *p* = 0.0074, *n* = 25). This correlation increased (r = 0.6; *p* = 0.0009, *n* = 25), when the antibody reactivity was directed to the COOH part of CXCL4 (anti-CXCL4-short). The antibody response to the CXCL4-L1-COOH portion also significantly correlated with IFN-a (anti-CXCL4-L1 short; r = 0.049, *p* = 0.0065, *n*= 25). We observed no correlations between anti-CXCL4/CXCL4-L1 autoantibodies and IFN-I in eaSSc and VEDOSS (wt CXCL4 eaSSc: r = 0.05, *p* = 0.1, *n* = 13; CXCL4-L1/IFN-I eaSSc: r = −0.05, *p* = 0.38, *n* = 32; CXCL4/IFN-I VEDOSS: r = −0.05, *p* = 0.40. *n* = 32; CXCL4-L1/IFN-I VEDOSS: r = 0.05, *p* = 0.39, *n* = 32).

Since we have shown that anti-CXCL4 antibodies were higher in patients with DU, as reported above, we wondered whether the patients with DU were also those exhibiting an IFN-I-signature. Five out of 13 (38%) of the SSc patients with DU had plasma IFN-a. In contrast, none of the patients without DU expressed IFN-a in their plasma. The difference in IFN-a expression between the two groups was significant (*p* = 0.02, Figure 3b, left panel).

Interestingly, the number of DU measured in the same lsSSc correlated with the presence, in plasma, of IFN-a (measured by ELISA assay, Figure 3b right panel). These results suggest that the capacity of anti-CXCL4 autoantibodies to amplify IFN-I production in SSc [15] is likely instrumental for DU formation, but only in lsSSc. These findings once again may indicate that the effector functions of the anti-CXCL4 antibodies are different in early disease, as compared to advanced disease.

### 3.4. IFN-I Expression, but Not Anti-CXCL4/CXCL4-L1 Antibody Reactivity, Differs in VEDOSS SSc-Progressors versus SSc-Non-Progressors

**Figure 4:**
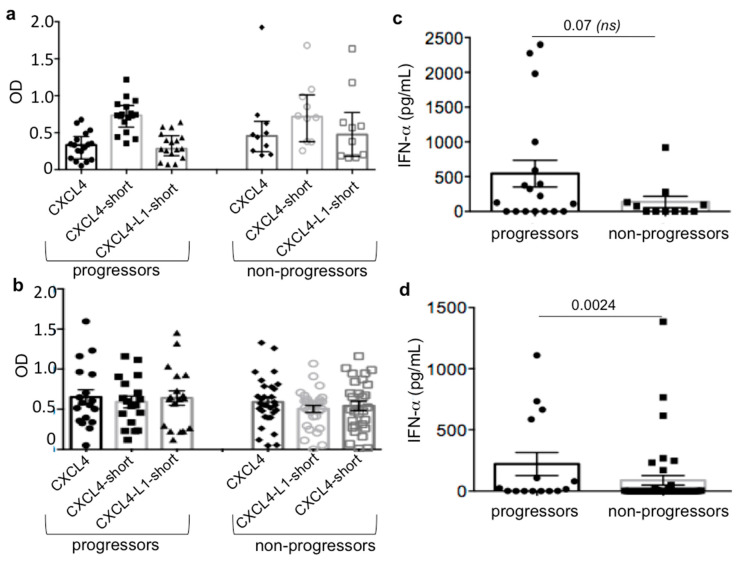
Anti-CXCL4/CXCL4-L1 autoantibodies do not discriminate SSc-progressors from SSc-non-progressors among VEDOSS. (**a**,**b**) Plasma of VEDOSS patients, divided in progressors and non-progressors, were tested for antibody reactivity to entire CXCL4 (CXCL4) or to the 27 mer peptides spanning the COOH-term of CXCL4 (CXCL4-short) or CXCL4-L1 (CXCL4-L1-short), by ELISA in the discovery VEDOSS cohort (**a**), and in the replication VEDOSS cohort (**b**). Results are reported as optical density (OD). (**c**,**d**) IFN-a was tested in plasma of VEDOSS patients of the discovery (**c**) and replication (**d**) cohort by ELISA. In the graphs, amounts of IFN-a are reported in comparison for progressors and non-progressors. In all graphs horizontal bars represent the means, vertical bars are standard error of the mean (SEM); *p* values by Mann–Whitney test.

To address whether the antibody autoreactivity to CXCL4/CXCL4-L1 could be associated with subsequent progression towards SSc in VEDOSS, we studied the VEDOSS patients prospectively. We identified and sorted the VEDOSS patients in SSc-progressors (*n* = 17) and SSc-non-progressors (*n* = 10). Unfortunately, the two groups showed a similar autoantibody response (Figure 4a). Anti-CXCL4/CXCL4-L1 autoantibodies were measured also in a replication VEDOSS cohort (Figure S4): The results obtained offered a similar picture, with no significant differences in SSc-progressors, (*n* = 15) versus SSc-non-progressors, (*n* = 33), with respect to the antibody reactivity to CXCL4/CXCL4-L1 (Figure 4b). Despite this, in both VEDOSS cohorts, the SSc-progressors tended to present a higher IFN-I signature (measured by ELISA test in plasma, discovery cohort, or sera, replication cohort) (Figure 4c,d). This difference was significant in the replication VEDOSS cohort (Figure 4d). Anti-CXCL4 autoantibodies did not correlate with IFN-I in the VEDOSS cohorts (VEDOSS disc. cohort: r = −0.018, *p* = 0.46, *n* = 32; SSc-progressors: r = −0.259, *p* = 0.11, *n* = 17; SSc-non-progressors: r = 0.13, *p* = 0.36, *n* = 10; VEDOSS repl. cohort: r = 0.15, *p* = 0.16, *n* = 48; SSc-progressors: r = −0.20, *p* = 0.21, *n* = 18; SSc-non-progressors: r = 0.005, *p* = 0.49, *n* = 30). These results indicate that anti-CXCL4 autoantibodies cannot discriminate between SSc progressors and non-progressors in VEDOSS. However, measurement of the IFN-I-signature may discriminate the SSc-progressor group, who showed a more frequent and higher IFN-α in blood. These results reinforce the assumption that anti-CXCL4 autoantibodies are qualitatively different between the first disease manifestations and late disease stages.

## 4. Discussion

In this study, we have corroborated our previous observations, indicating in CXCL4 a new SSc autoantigen [15]. Thus, anti-CXCL4 antibodies are really novel SSc biomarkers. In addition, we show that anti-CXCL4-L1 autoantibodies also behave as SSc biomarkers. Indeed, in the present work, we concomitantly addressed the capacity of SSc-autoantibodies to recognize CXCL4-L1, the non-allelic variant of CXCL4. It can be assumed that anti-CXCL4 autoantibodies cross-react to CXCL4-L1, as the two molecules differ only by three amino acid substitutions at their COOH-part. These substitutions occur at the amino acid residues 89, 97 and 98 [P → L at res. 89; K → E at res. 97; L → H at res. 98; see UniProtKB “(P02776 (PLF4_HUMAN))” and “P10720 (PF4V_HUMAN)” for CXCL4 and CXCL4-L1 sequences and amino acid residues number].

However, our assay suggests that, in lsSSc, a consistent portion of patients harbor autoantibodies exclusively reacting to the COOH-part of CXCL4-L1. As the COOH-peptides used in our ELISA assays span the last 27 amino acids of the COOH-part of CXCL4/CXCL4-L1, it can be argued that the autoantibodies defined “anti-CXCL4-L1-specific” recognize the peptide portion in common between CXCL4 and CXCL4-L1 (residues from amino acid 70 to 87, an 18 mer peptide). However, we consider this possibility unlikely, as the autoantibodies reacting to the COOH-part of CXCL4-L1 should be able to recognize also the COOH-part of the wt CXCL4, as well as the entire CXCL4, as both molecules contain the amino acid portion spanning residues 70–87). We are aware that conformational epitopes recognition by the autoantibodies may be the reason for the differential reactivity observed. It is indeed known that CXCL4 and CXCL4-L1 have a different conformational structure [29]. Whether this is also true for the COOH-portions of the two non-allelic variants is unclear, although this remains a possibility. Indeed, the two 27-mer peptides spanning the COOH-part of CXCL4 and CXCL4-L1 were shown to differ for their capacity to mediate anti-angiogenic effects [16,29]. It is interesting, though, that only long-lasting SSc patients show autoantibodies exclusively reacting to the CXCL4-L1 peptide. This may imply that the autoantibody response in lsSSc patients is qualitatively different from that detectable at early disease stages and even in VEDOSS. Epitope spreading over time can explain this finding. It is of course difficult to explain why some lsSSc only recognize CXCL4-L1. However, it could also be that the response to wt CXCL4 was present at early stages, and it deviated towards CXCL4-L1 at later stages. Indeed, CXCL4 is released by activated platelets together with CXCL4-L1, but if the disease is kept under control for some time, levels of the antigen CXCL4 could decline. Instead, CXCL4-L1 is constitutively expressed, and it may be hypothesized that smooth cells of blood vessels, which produce CXCL4-L1, could be the targets of the anti-CXCL4-L1 autoantibodies [16]. The importance of CXCL4-L1 in SSc is indeed the object of a future analysis.

The most striking difference in the autoantibody reactivity in lsSSc versus VEDOSS/eaSSc patients can perhaps be ascribed to possible alternative effector functions of these autoantibodies. We supposed this, due to the observation that the magnitude of the anti-CXCL4 autoantibody response positively correlates with IFN-I and DU, and other skin-involvement parameters in lsSSc but not in VEDOSS/eaSSc. In contrast, the correlation coefficients calculated between anti-CXCL4 reactivity and IFN-a blood levels, or DU numbers, tend to be negative in eaSSc and VEDOSS. A limitation in the correlation studies between anti-CXCL4 autoantibodies or IFN-a and DU may be that while antibodies and IFN-a are precisely measurable parameters, the DU counts may be subjective and do not take into accounts the extensions of each single lesion. Therefore, our Spearman correlations involving DU should be interpreted with caution. Nevertheless, we found that in the group of lsSSc patients with DU various patients showed detectable plasma levels of IFN-a, whereas none of the lsSSc patients that did not present DU had detectable IFN-a in their plasma. This reinforces the idea that anti-CXCL4 antibodies implement IFN-a levels, which in turn favor DU formation. Interestingly, anti-CXCL4 autoantibodies are significantly higher in patients with pitting scars in the lsSSc, but significantly lower in those with eaSSc. Since CXCL4 is highly up-regulated in eaSSc (especially in the diffuse form [7]), as well as in VEDOSS [30], one could speculate that the initial production of autoantibodies to CXCL4 could serve to neutralize CXCL4 excess, which may also block excess of IFN-I production at the beginning of the SSc symptoms. In this regard, we have shown that the levels of CXCL4-DNA complexes in eaSSc greatly correlates with amounts of IFN-I in blood [12]. Early anti-CXCL4 autoantibodies may attempt to neutralize the interferogenic effects of such complexes at the beginning of the disease. Here, we have seen that VEDOSS patients, that are SSc-progressors, express the highest IFN-I concentrations in their plasma/sera. Thus IFN-I may be deleterious for SSc-progression. Still, many SSc-progressors do not express IFN-I. This means that IFN-I is necessary but not sufficient to drive SSc-progression and does not represent a suitable SSc-progression marker. Despite the fact that we have not observed differences in anti-CXCL4-autoantibody reactivity in SSc-progressors versus non-progressors, it is still possible that the autoantibody specificity and effector functions evolve in a different manner in SSc-progressors versus non-progressors at a given time point. This deserves deeper investigations. We could have expected that anti-CXCL4 antibodies contributed differently to the IFN-I signature in SSc-progressors versus non-progressors, but correlation analyses do not clearly favor this view. However, the results also suggest that the factors that contribute to the SSc IFN-I-signature [31] in eaSSc cannot be the anti-CXCL4 autoantibodies. Major contributors could be rather the circulating CXCL4-DNA complexes [12], or other autoantibody specificities (ACA, ATA), as well as autoantibody specificities not yet elucidated [9,10].

Additional limitations of this study are a lack of definitive demonstration that autoantibodies to CXCL4 and/or CXCL4-L1 are really endowed with different (perhaps opposite) effector functions in late versus early disease. Indeed, we base these assumptions on correlation analyses and not on functional assays. Functional assays require isolation of autoantibodies from many SSc patients and appropriate in vitro test and controls. However, we believe that the present findings could be a starting point to stimulate discussion and research on these aspects, which may lead to the discovery of new pathogenic mechanisms in SSc, and elucidate a role for anti-CXCL4/CXCL4-L1 antibodies as disease biomarkers.

## 5. Conclusions

The results of this study confirm the presence of autoantibodies to CXCL4 in SSc patients.

They extend these previous findings, via identifications of the same autoantibody specificity in VEDOSS.

We additionally report previously unappreciated correlations of anti-CXCL4 and/or anti-CXCL4-L1 autoantibodies with skin characteristics that can be related to each other (calcinosis, pitting scares and DU) in addition to lung fibrosis, which is also confirmed [15].

Our results also show for the first time that the CXCL4-L1 variant can be considered an additional autoantigen in SSc.

## Figures and Tables

**Table 1:** Main clinical, Demographic and Laboratory features of SSc, HD and VEDOSS patients at baseline.

Main Clinical, Demographic and Laboratory Parameters	SSc (*n* = 42)	VEDOSS1 (*n* = 31) (Discovery Cohort)	VEDOSS2 (*n* = 48) (Replication Cohort)	*p* Values VEDOSS1 vs. VEDOSS2	HD (*n* = 25)
Age, mean (range): years	52.5 (32–71)	50 (26–61)	47 (25–70)	ns	48 (29–57)
Sex (M/F):	1/41	0/31	2/46	ns	10/15
Disease duration from 1st visit (months)(range)	74.4 (12–252)	135.6 (36–504)	120 (36–500)	ns	N/A
SSc Form (limited/diffuse)	1/41	N/A	N/A	N/A	N/A
Ea lim/ea diffuse	0/14	N/A	N/A	N/A	N/A
mRSS (mean, range)	16.6 (6–36)	N/A	N/A	N/A	N/A
ACA positivity	5%	64%	61%	ns	N/A
ATA positivity	71%	23%	5%	*p* = 0.04	N/A
aRNAP3 positivity	14%	3%	-	N/A	N/A
Calcinosis	50%	0%	2.5%	ns	N/A
Pitting scars	60%	0%	-	N/A	N/A
Raynaud Phenomenon	93%	100%	97%	ns	N/A
DU	50%	0%	0%	ns	N/A
Teleangectasia	71%	0%	5%	ns	N/A
Pulm Art. Hypertension	25%	0%	8%	ns	N/A
Lung fibrosis (%)	33%	0%	0%	ns	N/A
DLCO (%) (mean)	65.7%	85.6%	85.5%	ns	N/A
DLCO < 80%	88%	38%	26%	*p* = 0.001	N/A
Gastroint. Involv.	0%	0%	0%	ns	N/A
Synovitis	0%	0%	0%	ns	N/A
Sclerodactilia	69%	-	-	N/A	N/A
DMARDs	99%	16%	18%	ns	N/A

Legend: Ea SSc, early diffuse SSc; ACA, anti-centromers antibodies; ATA, anti-topoisomerase antibodies; aRNAP3, anti-RNA-polimerase 3; DU, digital ulcers; DLCO, Diffusion Lung CO; DMARDS, Disease modifying antirheumatic drugs. *p*, significant differences between the main and replication VEDOSS cohorts analyzed (Mann–Whitney test); ns = non-significant difference. N/A, not applicable, “-”, data not available.

## Data Availability

Data relative to this work are available upon reasonable request to the corresponding author L.F.

## Supplementary Materials

The following are available online at https://www.mdpi.com/article/10.3390/antib10020012/s1. Figure S1: In eaSSc there is a negative correlation between anti-CXCL4 antibodies and DU; Figure S2: LsSSc with lung fibrosis have higher anti-CXCL4 antibodies; Figure S3: EaSSc with pitting scars have lower anti-CXCL4 antibodies then patients with not pitting scars; Figure S4: Anti-CXCL4 antibodies are present in the VEDOSS replication cohort.
