# Supplementary material for: New Autoantibody Specificities in Systemic Sclerosis and Very Early Systemic Sclerosis
Source: Antibodies (Basel). 2021 Mar 28;10(2):12. doi: 10.3390/antib10020012 (PMC8103273; doi:10.3390/antib10020012)
Supplement: Supplementary file 1 [file antibodies-10-00012-s001.pdf]

SUPPLEMENTARY FIGURES

New Autoantibody Specificities in Systemic Sclerosis and Very Early Systemic Sclerosis

Roberto Lande, Raffaella Palazzo, Anna Mennella, Immacolata Pietraforte, Marius Cadar, Katia Stefanantoni, Curdin Conrad, Valeria Riccieri [4] and Loredana Frasca

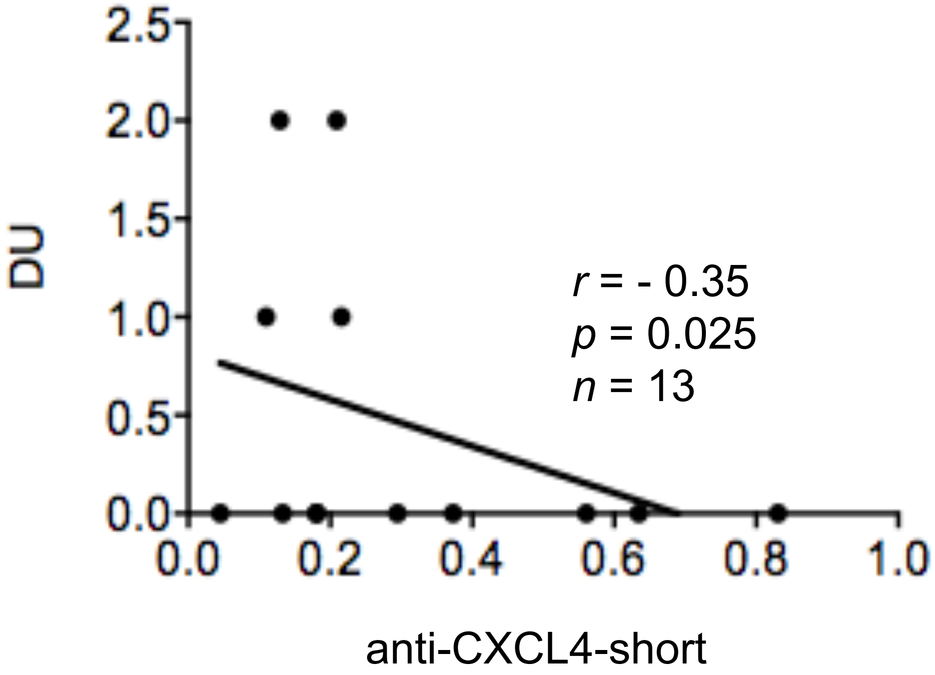

Figure S1. In eaSSc there is a negative correlation between anti-CXCL4 antibodies and DU. Number of DU in eaSSc plotted against anti-CXCL4 antibodies directed to the COOH-term of CXCL4. *r* Spearman coefficient, *p* values and sample size *n* indicated.

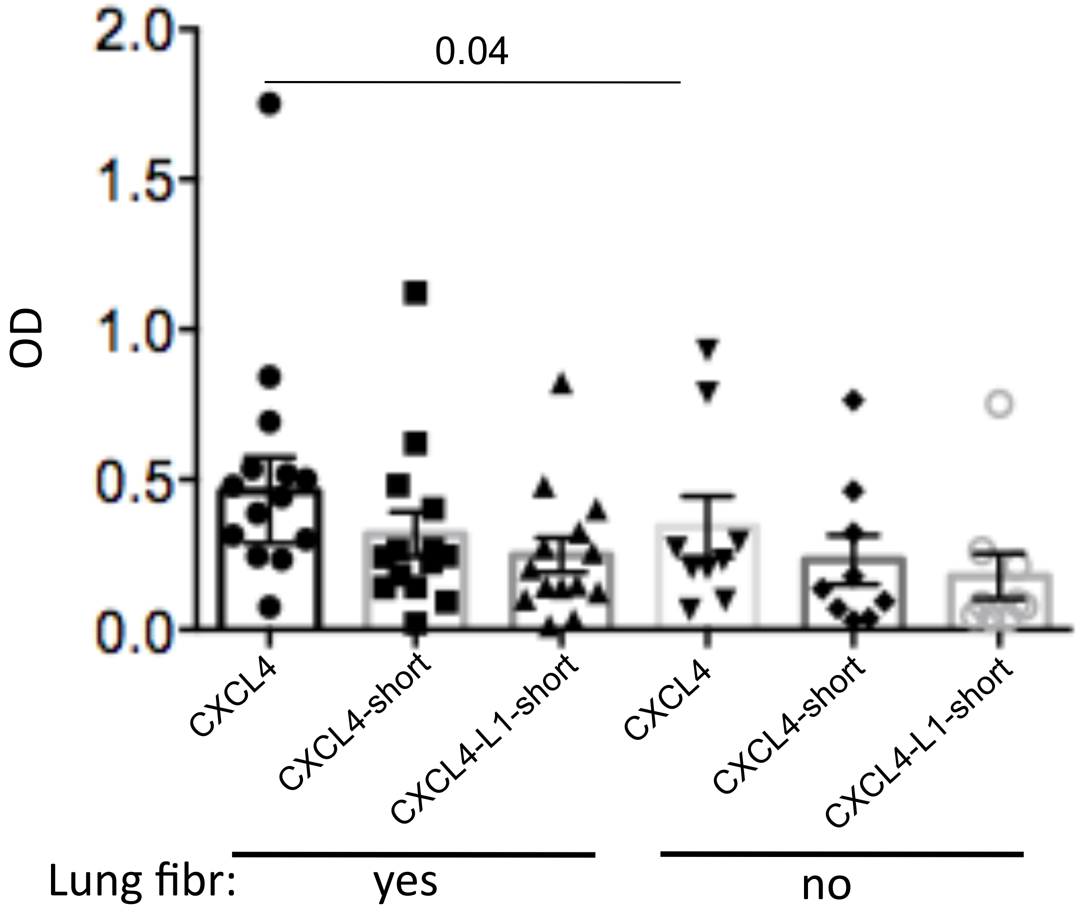

Figure S2. LsSSc with lung fibrosis have higher anti-CXCL4 antibodies. Patients with lung fibrosis and without lung fibrosis were tested for antibody reactivity to entire CXCL4 (CXCL4) or to the 27 mer peptides spanning the COOH-term of CXCL4 (CXCL4-short) or CXCL4-L1 (CXCL4-L1-short), by ELISA. Results are reported as optical density (OD). Horizontal bars represent the means, vertical bars are standare error of the mean (SEM), *p* values by Mann-Whitney test.

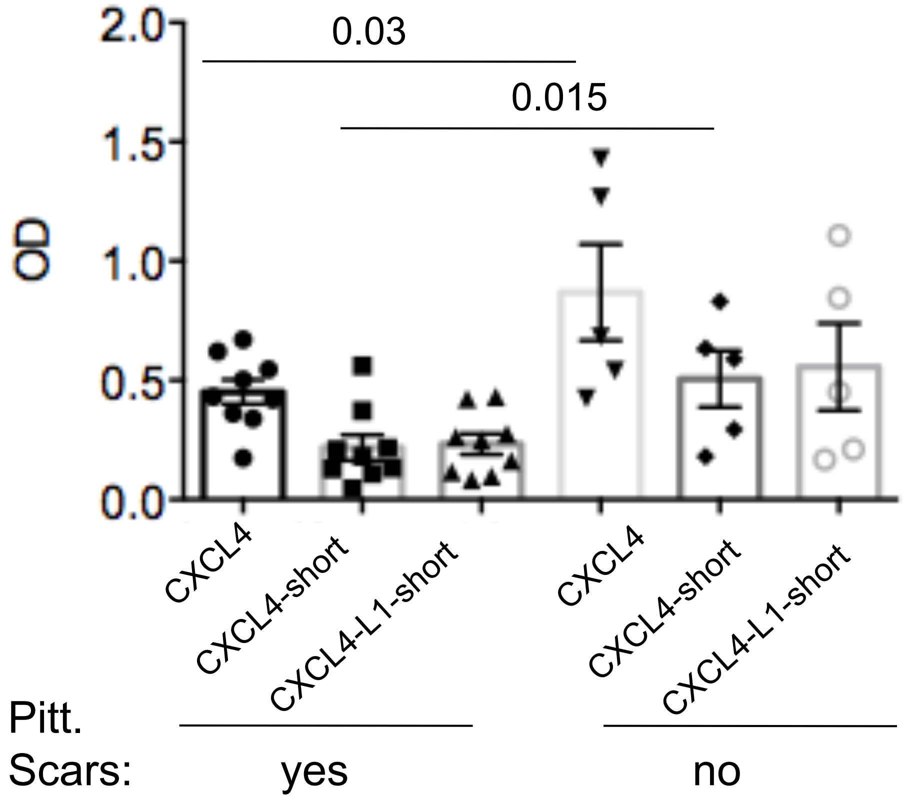

Figure S3. EaSSc with pitting scars have lower anti-CXCL4 antibodies then patients without pitting scars. Patients with pitting scars and without pitting scars were tested for antibody reactivity to entire CXCL4 (CXCL4) or to the 27 mer peptides spanning the COOH-term of CXCL4 (CXCL4-short) or CXCL4-L1 (CXCL4-L1-short), by ELISA. Results are reported as optical density (OD). Horizontal bars represent the means, vertical bars are standare error of the mean (SEM), *p* values by Mann-Whitney test.

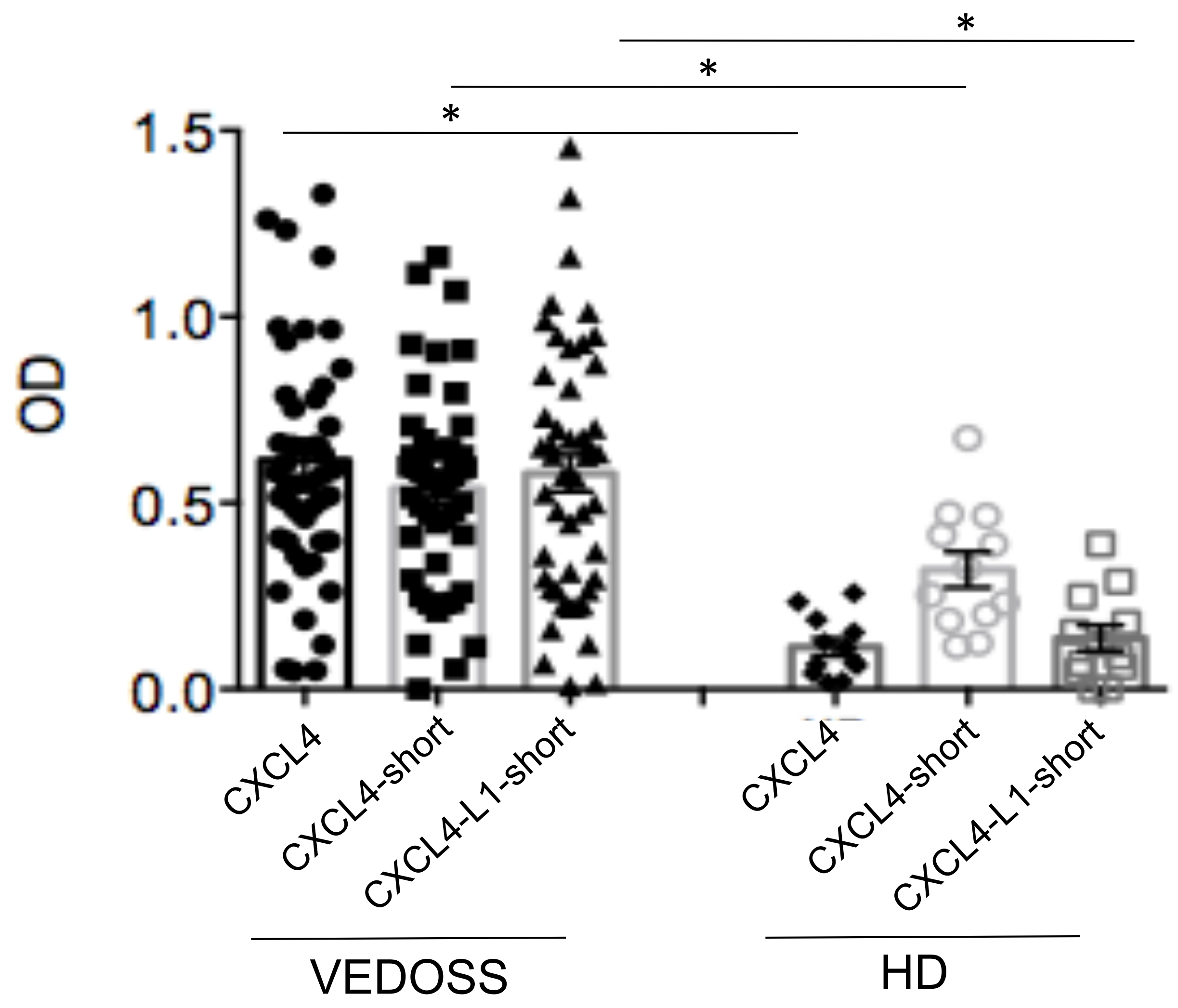

Figure S4. Anti-CXCL4 antibodies are present in the VEDOSS replication cohort. Sera of VEDOSS patients and HD were tested for antibody reactivity to entire CXCL4 (CXCL4) or to the 27mer peptides spanning the COOH-term of CXCL4 (CXCL4-short) or CXCL4-L1 (CXCL4-L1-short), by ELISA. Results are reported as optical density (OD). Horizontal bars represent the means, vertical bars are standare error of the mean (SEM), P values by Mann-Whitney test. * $p < 0.05$
